# Comment on *Timely deposition of macromolecular structures is necessary for peer review* by Joosten *et al.* (2013)

**DOI:** 10.1107/S0907444913029168

**Published:** 2013-11-19

**Authors:** Helen Berman, Gerard J. Kleywegt, Haruki Nakamura, John L. Markley

**Affiliations:** a Department of Chemistry & Chemical Biology, Center for Integrative Proteomics Research, Rutgers, The State University of New Jersey, 174 Frelinghuysen Road, Piscataway, NJ 08854, USA; b PDBe, European Molecular Biology Laboratory–European Bioinformatics Institute, Cambridge CB10 1SD, UK; c PDBj, Institute for Protein Research, Osaka University, 3-2 Yamadaoka, Suita, Osaka, 565-0871, Japan; d BioMagResBank, Department of Biochemistry, University of Wisconsin-Madison, Madison, WI 53706, USA

**Keywords:** wwPDB, deposition, macromolecular data

## Abstract

The wwPDB responds to the article by Joosten *et al.* [(2013), *Acta Cryst. *D**69**, 2293–2295].

## Abstract 2

A response to the article by Joosten *et al.* [(2013), *Acta Cryst. *D**69**, 2293–2295].

The Worldwide Protein Data Bank (wwPDB) strongly agrees with the overall views expressed by Joosten *et al.* (2013 ▶) in their article about timely deposition of macromolecular structures in the Protein Data Bank. In 2010, *Acta Crystallographica Section D* began to require validation reports as part of the manuscript-submission process. In that same year, the wwPDB sent letters to the key journals that publish structures requesting that they require authors to submit wwPDB validation reports at the same time as their manuscripts. In this way, reviewers are able to better evaluate the work. The *Journal of Biological Chemistry*, which is currently the journal that publishes the largest number of papers per year about structures of biological macromolecules, began requiring these reports in 2012.

Joosten *et al.* suggest that it would be helpful to have an option to suppress entry titles at the time of submission to the PDB until the structure is released. Policy matters such as this are regularly reviewed by the wwPDB partners and its Advisory Committee (wwPDB AC). The issue was discussed at our 2013 meeting, and it was agreed that we will make this option available in the new wwPDB Deposition Tool that will be launched early in 2014.
